# The impact mechanism of green exercise on college students' suicidal ideation: a mediation analysis based on entrance anxiety

**DOI:** 10.3389/fpsyg.2026.1735347

**Published:** 2026-03-31

**Authors:** Mingmin Kong, Bo Peng, Weisong Chen, Tong Liu, Zhongen Yin

**Affiliations:** 1 School of Sports Training, Chengdu Sport University, Chengdu, Sichuan, China; 2 College of Physical Education and Health Management, Chongqing University of Education, Nan'an, Chongqing, China

**Keywords:** college freshmen, entrance anxiety, green exercise, mediation analysis, structural equation modeling, suicidal ideation

## Abstract

**Purpose:**

This study aimed to examine the relationship between green exercise and suicidal ideation among first-year university students and to explore the mediating role of entrance anxiety in this association.

**Methods:**

A total of 713 freshmen participated in a cross-sectional survey using standardized questionnaires assessing green exercise, entrance anxiety, and suicidal ideation. Demographic variables (gender, family economic status, place of origin, and only-child status) were controlled. Data were analyzed with SPSS 27.0 and AMOS 26.0. Reliability, validity, and common method bias were tested, followed by structural equation modeling (SEM) and a bias-corrected bootstrapping analysis with 5,000 resamples to examine direct and indirect effects.

**Results:**

The SEM results indicated that green exercise significantly reduced entrance anxiety (β = −0.36, *p* < 0.001) and suicidal ideation (β = −0.42, *p* < 0.001), whereas entrance anxiety positively predicted suicidal ideation (β = 0.40, *p* < 0.001). Bootstrapping confirmed that entrance anxiety partially mediated the relationship between green exercise and suicidal ideation. The total effect of green exercise on suicidal ideation was significant (β = −0.57, *p* < 0.001), including a direct effect (β = −0.42, 73.68%) and an indirect effect through entrance anxiety (β = −0.15, 26.32%), with confidence intervals excluding zero.

**Conclusion:**

Green exercise effectively alleviates entrance anxiety and reduces suicidal ideation among university freshmen. These findings highlight the mental health benefits of outdoor physical activity and suggest that integrating green exercise into campus life may serve as an effective preventive strategy against anxiety and suicidal ideation.

## 1 Introduction

College students are in a critical transitional period from dependence on the campus environment to independent participation in society. Within a relatively short time, they must adjust their learning styles, rebuild interpersonal relationships, initiate career planning, and renegotiate their self-identity. As a result, they are more likely to be exposed to multiple stressors, including academic demands, interpersonal pressures, financial burdens, and uncertainty about the future, making them a population with concentrated mental health risks. In recent years, mental health problems among college students have shown an increasing trend, and suicidal ideation, as a key antecedent in the chain of suicidal behaviors, has become an important hidden danger to students' psychological safety and public health (Klonoff-Cohen et al., 2024). Suicidal ideation not only serves as a critical signal for identifying potential suicide risk but is also one of the core indicators for psychological crisis assessment and early intervention (Huang et al., 2024). Previous studies have indicated that the development of suicidal ideation is closely associated with individuals' negative emotional experiences, anxiety levels, stress-coping capacities, and social adaptation status (Wen et al., 2024). Therefore, clarifying the mechanisms that influence college students' suicidal ideation and exploring scalable protective factors are of substantial theoretical and practical significance for promoting mental health and preventing suicide in higher education settings.

Against the backdrop of increasingly in-depth research on suicide risk, the fields of health psychology and sport psychology have in recent years extended their focus to physical activity in natural environments. With the introduction of the concept of “green exercise,” researchers have emphasized that engaging in physical activities in natural settings (e.g., outdoor running, cycling, hiking, gardening, or exercising in campus green spaces) may offer mental health benefits distinct from those of general exercise (Loureiro and Veloso, 2017). Existing evidence suggests that green exercise not only contributes to improved cardiopulmonary function and overall physical health but is also associated with reductions in negative emotional states such as anxiety, depression, and stress responses, as well as increases in positive emotional experiences (Twohig-Bennett and Jones, 2018; Lahart et al., 2019). From a mechanistic perspective, green exercise may produce greater psychological relaxation and enhanced subjective wellbeing than indoor exercise through the attentional restoration and emotional recovery effects of natural landscapes, physiological arousal regulation brought about by environmental exposure (e.g., better air quality and soothing sensory experiences), and increased opportunities for social interaction and support in outdoor contexts (Gladwell et al., 2013). For college students, green exercise is typically characterized by low cost, high accessibility, and strong acceptability, and some studies have regarded it as a potential intervention that integrates physical fitness enhancement with emotion regulation functions (Coventry et al., 2021; Bramwell et al., 2023). However, it should be noted that although the relationship between green exercise and general mental health indicators (e.g., stress, depression, and anxiety) has been widely discussed, the association between green exercise and suicidal ideation—a high-risk outcome variable—still lacks systematic, direct, and mechanism-oriented empirical testing, and the quantity of related research and the strength of the evidence base remain insufficient.

Among the various risk factors influencing college students' suicidal ideation, entrance anxiety represents an important source of context-specific psychological stress. Entrance anxiety refers to the tension, worry, and discomfort commonly experienced by first-year students during the initial stage of university due to changes in the learning environment, increased academic demands, reconstruction of social support systems, and role transitions (Jones, 2024). Moderate anxiety may facilitate alertness and adjustment, but excessive or persistent entrance anxiety is often associated with adverse outcomes such as sleep disturbances, low mood, social withdrawal, and difficulties in academic adaptation (Li et al., 2024). Studies have found that college students with higher anxiety levels are more likely to experience self-denial, helplessness, and negative cognitive processing, thereby increasing the likelihood of suicidal ideation (Park et al., 2023; Asfaw et al., 2020). Meanwhile, exercise behaviors are widely considered an effective approach to alleviating anxiety (Han et al., 2025). Exercise may improve emotional states and stress responses by regulating the hypothalamic-pituitary-adrenal (HPA) axis and promoting the secretion of neurotransmitters such as endorphins, and it may also reduce anxiety by enhancing self-efficacy, improving sleep, and strengthening perceived control (Zhang and Kong, 2025; Ahindru et al., 2023). Based on this, it can be inferred that green exercise—an activity pattern combining the benefits of exercise with the advantages of natural environment exposure—may exert a stronger buffering effect on entrance anxiety and, in turn, influence suicidal ideation. Nevertheless, research on the chain mechanism of “green exercise → entrance anxiety → suicidal ideation” remains relatively scarce, especially in samples of first-year students or those in the entrance adjustment period, and consistent and clear empirical evidence has yet to be established.

In summary, existing research provides an important foundation for understanding the mental health benefits of green exercise and the risk implications of entrance anxiety, but several gaps remain. First, green exercise studies have largely focused on general mental health indicators, with insufficient attention to suicidal ideation as a high-risk psychological outcome. Second, pathway-oriented research on how green exercise influences psychological risk is limited, and there is a lack of mechanism testing centered on context-specific stress variables such as entrance anxiety. Third, although green exercise is highly feasible and scalable in university settings, evidence for its role in suicide prevention and risk reduction remains inadequate. Based on these theoretical gaps, the present study incorporates entrance anxiety into the framework linking green exercise and suicidal ideation, aiming to develop a more explanatory mechanism model. The innovations of this study are mainly reflected in: (1) extending the outcome variable by shifting the research focus from general mental health indicators to suicidal ideation, a higher-risk and more public-health-relevant outcome; (2) clarifying the mechanism pathway by introducing entrance anxiety as a mediating variable to test whether green exercise may indirectly reduce suicidal ideation by alleviating anxiety during the entrance adjustment period; and (3) strengthening applied value by emphasizing a feasible behavioral intervention in university contexts (green exercise participation) and providing more actionable evidence for campus sports program design and mental health education.

Accordingly, this study aims to examine the relationship between college students' participation in green exercise and suicidal ideation and to test the mediating role of entrance anxiety in this association. The specific research questions are: (1) Is participation in green exercise significantly associated with lower levels of suicidal ideation? (2) Does entrance anxiety mediate the relationship between green exercise and suicidal ideation? Data will be collected through a questionnaire survey, and structural equation modeling will be used to test the proposed theoretical pathways. The findings are expected to reveal the potential protective role of green exercise in reducing psychological risk among college students from both behavioral and psychological mechanistic perspectives, thereby providing scientific evidence for mental health promotion in higher education, the allocation of campus green space and sports resources, and early intervention strategies for suicide prevention.

## 2 Literature review and hypotheses

### 2.1 Psychological mechanisms and influencing factors of suicidal ideation

Suicidal ideation refers to thoughts or cognitions about ending one's own life and is an early warning sign of suicidal behavior (Klonsky et al., 2021). According to Beck's cognitive vulnerability model, the formation of suicidal ideation often stems from the accumulation of negative self-schemas, feelings of helplessness, and hopelessness (Beck et al., 1993). When individuals remain in prolonged states of anxiety, depression, or emotional deprivation, and lack effective social support and coping resources, these can develop into persistent suicidal ideation (Howlader et al., 2024; Hu et al., 2023).

Among college students, the risk of suicidal ideation is particularly prominent. The start of independent living, academic and interpersonal pressures, and uncertainty about future careers can all serve as triggers for psychological crises (Cecchin et al., 2024; Okechukwu et al., 2022). Studies show that adaptive anxiety, loneliness, and cognitive distortions during the initial phase of college entry are important psychological variables predicting suicidal ideation (Satyander Kumar, 2024; Hunt and Eisenberg, 2010; Hager et al., 2022). From a macro perspective, the formation of suicidal ideation reflects the “stress-vulnerability” interaction model, wherein external stressors and internal psychological vulnerability interact, leading to emotional imbalance and a chain of negative thoughts (Van Heeringen, 2012; Steinberg and Mann, 2020). Therefore, exploring psychological and environmental factors, such as exercise, contact with nature, and social belonging, that can buffer this stress process is an important pathway for understanding and intervening in college students' suicidal ideation.

### 2.2 Research progress on green exercise and mental health

Green exercise refers to physical activities conducted in natural or semi-natural environments, including outdoor running, hiking, cycling, gardening, and fitness activities in campus green spaces (Loureiro and Veloso, 2017). The concept was first introduced by Pretty et al. (2003), who emphasized the “synergistic health effects” of combining natural environments with physical activity (Pretty et al., 2003). A large number of studies have verified the positive effects of green exercise from physiological, psychological, and social perspectives: Physiological: Green exercise can alleviate physiological stress responses by regulating sympathetic nervous activity, lowering cortisol levels, and improving heart rate variability (Aras et al., 2024; De Brito et al., 2020). Psychological: According to Attention Restoration Theory, natural environments help individuals recover from prolonged cognitive load and emotional tension, promoting positive emotions and psychological relaxation (Kaplan, 1995). Social: Green exercise often occurs in open spaces, where interaction among participants enhances social connection and support, thereby increasing psychological belonging and wellbeing (Barton and Pretty, 2010; Pretty et al., 2005).

Empirical research has shown that green exercise significantly reduces anxiety, depression, tension, and fatigue, while also improving self-esteem, attention, and subjective wellbeing (Bratman et al., 2019; Thompson Coon et al., 2011). Some studies have found that even short-term exposure to nature (such as 10 min of outdoor activity) can significantly reduce heart rate and negative emotions (Lahart et al., 2019; Wicks et al., 2022). However, the potential impact mechanism of green exercise on suicidal ideation has not been systematically verified. Some studies have indirectly suggested that contact with nature can reduce emotional distress and rumination (Bratman et al., 2015, 2021), possibly by emotional repair and stress buffering, which may indirectly reduce the risk of suicide, but this hypothesis still lacks empirical support in college student samples.

### 2.3 The role of college entry anxiety in college students' mental health

College entry anxiety is a comprehensive stress response faced by new students after entering a new environment, manifested as worry about the unknown environment, fear of adaptation failure, and sensitivity to social evaluation (Jones, 2024). Its core psychological mechanism can be explained by Lazarus and Folkman (1984) stress-coping theory: when individuals perceive that environmental demands exceed their psychological resources or coping abilities, anxiety and discomfort arise. Studies have found that college entry anxiety is significantly related to poor academic adaptation, social withdrawal, and decreased self-efficacy, and may also trigger depression and suicidal ideation (Yang et al., 2023; He et al., 2025). Individuals in a prolonged high-anxiety state are more likely to experience negative attribution and cognitive rigidity, thus magnifying self-denial and hopelessness (Saricali et al., 2022).

On the other hand, physical exercise is considered an effective way to reduce anxiety (Anderson and Shivakumar, 2013; Henriksson et al., 2022). Exercise anxiety intervention theory posits that exercise can alleviate anxiety levels by physiological activation, neurotransmitter regulation (such as serotonin and endorphins), and enhancing self-efficacy (Craft and Perna, 2004). Particularly, green exercise, combining the dual effects of contact with nature and physical activity, has been shown to have a stronger alleviating effect on anxiety (Mackay and Neill, 2010). Therefore, it is reasonable to infer that green exercise may indirectly reduce college students' suicidal ideation by lowering college entry anxiety.

### 2.4 Theoretical model

Based on the literature review above, this study will propose the following hypotheses:

H1: Green exercise has a negative impact on suicidal ideation in university students.

H2: Green exercise has a negative impact on entrance anxiety.

H3: Entrance anxiety mediates the relationship between green exercise and suicidal ideation.

**Figure 1:**
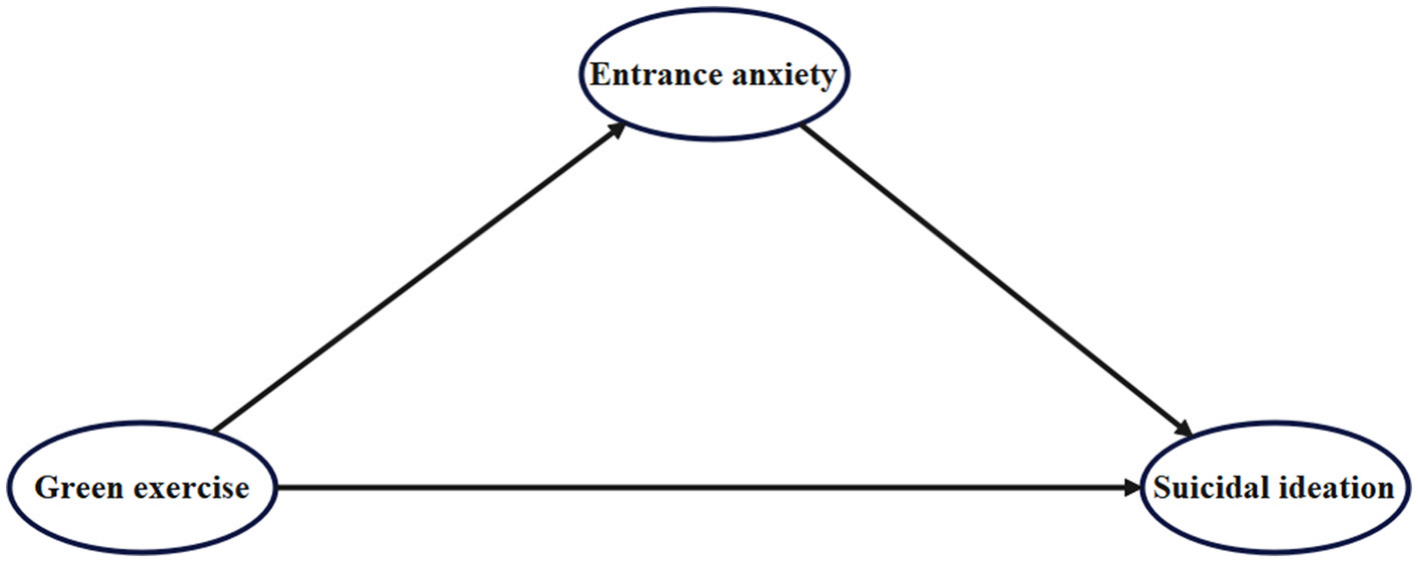
Theoretical model of the impact of green exercise on college students' suicidal ideation: the mediating effect of entrance anxiety.

The proposed hypotheses are integrated into a comprehensive theoretical framework (see Figure 1), aiming to explore the following aspects:

(1) Direct relationship: The direct impact of green exercise on college students' suicidal ideation.
(2) Relationship between independent and mediating variables: The effect of green exercise on entrance anxiety.
(3) Mediating effect: The mediating role of entrance anxiety in the relationship between green exercise and suicidal ideation.

This model hypothesizes that green exercise indirectly affects college students' suicidal ideation through entrance anxiety. Entrance anxiety is expected to play a crucial mediating role in this process, facilitating the impact of green exercise on suicidal ideation. By integrating these factors, the theoretical framework of this study provides a systematic perspective on the multidimensional influence mechanism of suicidal ideation among college students. Furthermore, it offers theoretical support and practical guidance for developing effective mental health intervention strategies to help reduce the suicide risk among college students.

## 3 Materials and methods

### 3.1 Research participants and data

This study employed a stratified random sampling method, selecting first-year students from universities in Sichuan, Chongqing, Guizhou, and Yunnan provinces and municipalities in China as participants. According to the empirical rule in social science research, the sample size is typically required to be 10 to 15 times the total number of questionnaire items (Jeffrey and Hoogland, 1998). The measurement tools used in this study include the Green Exercise Scale (5 items), the Entrance Anxiety Scale (7 items), and the Suicidal Ideation Scale (14 items), totaling 26 items. Accordingly, the estimated sample size ranged from 200 to 390 participants (26 items × 10 to 26 items × 15). This estimation method ensures the scientific rigor and coverage of the sample size design while meeting the statistical requirements of the study.

Data collection lasted from September 16, 2024, to March 31, 2025, during which a total of 800 questionnaires were distributed. A total of 713 valid questionnaires were collected, resulting in an effective response rate of 89.13%. During the questionnaire collection process, invalid questionnaires, such as those with missing data, inconsistent answers, or fixed-pattern responses, were excluded to ensure the validity and reliability of the data.

### 3.2 Measurement tools

All the measurement tools used in this study were based on widely validated scales that have demonstrated good reliability and validity, ensuring accurate assessment of the core characteristics of the relevant variables. The detailed information on the measurement tools is as follows:

Green exercise: green exercise was assessed using the green exercise scale developed by Wood et al. (2019). The scale consists of five items designed to assess the frequency, duration, and subjective perception of the impact of green exercise on psychological and physical health. The scale uses a five-point Likert scale (1 = “strongly disagree,” 5 = “strongly agree”), with higher scores indicating greater participation in green exercise. This scale has demonstrated good reliability and validity across different cultural contexts and populations (Cronbach's α > 0.80). In this study, the Cronbach's α coefficient for the Green Exercise Scale was 0.86, indicating high internal consistency.

Entrance anxiety: entrance anxiety was measured using the entrance anxiety scale developed by Huang Y. et al. (2024). The scale consists of seven items, designed to assess the anxiety and adaptation stress experienced by individuals when entering university. The scale uses a five-point Likert scale (1 = “strongly disagree,” 5 = “strongly agree”), with higher scores indicating greater entrance anxiety. Previous research has shown that this scale has good cross-cultural applicability and internal consistency (Cronbach's α > 0.80). In this study, the Cronbach's α coefficient for the Entrance Anxiety Scale was 0.89, indicating high internal consistency.

Suicidal ideation: suicidal ideation was assessed using the suicidal ideation scale developed by Osman et al. (1998). The scale includes 2 dimensions (active suicidal ideation, passive suicidal ideation) with a total of 16 items, designed to comprehensively assess individuals' suicidal thoughts and behavioral tendencies in the face of stress, challenges, or adversity. The scale uses a five-point Likert scale (1 = “strongly disagree,” 5 = “strongly agree”), with higher scores indicating stronger suicidal ideation. This scale has been validated across different cultural contexts and populations, demonstrating good reliability and validity (Cronbach's α > 0.80). In this study, the Cronbach's α coefficient for the Suicidal Ideation Scale was 0.91, indicating high internal consistency.

### 3.3 Data analysis

All statistical analyses were performed using IBM SPSS Statistics 27.0 and AMOS 26.0. Prior to formal analyses, the dataset was screened for missing values, normality, and outliers to ensure data integrity. All variables met the assumptions required for subsequent parametric analyses.

Descriptive statistics, including means, standard deviations, and frequency distributions, were first calculated to describe the demographic and main study variables. To evaluate the reliability of each scale, Cronbach's α coefficients were computed, with values above 0.70 considered acceptable, indicating adequate internal consistency (Ahmad et al., 2024). In addition, composite reliability (CR) and average variance extracted (AVE) were calculated for each latent construct to further assess measurement stability and convergent validity; CR values exceeding 0.70 and AVE values above 0.50 were used as judgment criteria (Cheung et al., 2024).

The measurement validity of the constructs was further examined using confirmatory factor analysis (CFA) in AMOS 26.0. Following conventional guidelines, standardized factor loadings greater than 0.60 were considered adequate, while acceptable model fit was determined using multiple indices: χ^2^/df < 3, CFI and TLI > 0.90, SRMR < 0.08, and RMSEA < 0.05 with its 90% confidence interval (Hair, 2011).

To test for potential common method bias, Harman's single-factor test and a series of CFAs were conducted. The presence of substantial bias would be indicated if a single factor explained more than 40% of the total variance or if the single-factor model exhibited poor fit relative to the multi-factor model (Kock et al., 2021).

After verifying the measurement quality, Pearson's correlation analysis was performed to examine the bivariate relationships among the key variables—green exercise, entrance anxiety, and suicidal ideation (Tang and Wen, 2020).

To further test the hypothesized relationships, hierarchical multiple regression analysis was employed. In Model 1, demographic variables (gender, family economic status, place of origin, and only-child status) were entered as controls; in Model 2, green exercise was added as the independent variable; and in Model 3, entrance anxiety was included as a mediator. The statistical significance of regression coefficients was evaluated at *p* < 0.05, *p* < 0.01, and *p* < 0.001 levels.

Finally, structural equation modeling (SEM) was conducted in AMOS 26.0 to examine both direct and indirect effects among the latent variables. Model adequacy was assessed using the same goodness-of-fit criteria described above. The mediating effect of entrance anxiety was further tested using a bias-corrected bootstrap method with 5,000 resamples, and mediation was considered significant if the 95% confidence interval (CI) for the indirect effect did not include zero (Preacher and Hayes, 2008).

## 4 Result

### 4.1 Sample characteristics

Table 1 presents the demographic composition of the participants. A total of 713 valid questionnaires were collected from first-year university students. The gender distribution was balanced, with 338 males (47.47%) and 375 females (52.59%). Regarding family economic status, 40.53% of respondents reported an *average* level, 24.54% *wealthy*, 30.43% *poor*, and 4.49% *very poor*. In terms of place of origin, 18.65% of the students came from *rural* areas, 50.07% from *towns*, and 31.28% from *cities*. With respect to only-child status, 49.09% of respondents were only children, and 50.91% had siblings. Overall, the demographic profile demonstrates a well-balanced and representative sample in terms of gender, socioeconomic background, and residential origins, which ensures the robustness of subsequent statistical analyses.

**Table 1:** Demographic characteristics of the sample (*N* = 713).

Variable	Category	Frequency	Percentage (%)
Gender	Male	338	47.47
	Female	375	52.59
Family economic status	Wealthy	175	24.54
	Average	289	40.53
	Poor	217	30.43
	Very poor	32	4.49
Place of origin	Rural	133	18.65
	Town	357	50.07
	City	223	31.28
Only-child status	Yes	350	49.09
	No	363	50.91

### 4.2 Descriptive statistics and measurement reliability

Table 2 summarizes the descriptive statistics, internal consistency reliability, and confirmatory factor analysis (CFA) results for the key study variables, including green exercise, entrance anxiety, and suicidal ideation. All three scales demonstrated satisfactory internal consistency. The composite reliability (CR) values (0.85–0.94) were all above 0.70, confirming the stability of the constructs. The average variance extracted (AVE) values (0.54–0.55) surpassed the 0.50 threshold, indicating adequate convergent validity. Factor loadings from the CFA ranged between 0.68 and 0.84 across all items, meeting the recommended level (>0.60). The measurement results collectively demonstrate that the latent constructs are reliable and valid for further hypothesis testing.

**Table 2:** Descriptive statistics, internal consistency reliability, and fit indices for confirmatory factor analysis (CFA) of key variables.

Variable	Mean	SD	Factor loading	CR	AVE
Green exercise	16.89	3.96	0.70–0.81	0.85	0.54
Entrance anxiety	20.84	5.60	0.71–0.83	0.89	0.54
Suicidal ideation	39.72	9.13	0.68–0.84	0.94	0.55

SD, standard deviation; CR, composite reliability; AVE, average variance extracted.

### 4.3 Common method bias test

To assess potential common method bias, Harman's single-factor test and confirmatory factor analyses (CFAs) were conducted. The unrotated exploratory factor analysis extracted four factors with eigenvalues greater than 1, accounting for a total of 61.30% of the variance. The first factor explained 33.59% of the total variance, which is below the critical threshold of 40%, indicating that common method bias was not a serious concern in this study.

As shown in Table 3, the one-factor model demonstrated poor fit (χ^2^/df = 18.59, CFI = 0.68, TLI = 0.63, SRMR = 0.142, RMSEA = 0.157 [0.150, 0.164]), indicating that the data cannot be explained by a single common latent factor. The three-factor model, in which each construct loaded on its corresponding latent variable (green exercise, entrance anxiety, and suicidal ideation), exhibited excellent model fit (χ^2^/df = 0.82, CFI = 1.00, TLI = 1.00, SRMR = 0.017, RMSEA = 0.001 [0.001, 0.012]). Because this model was saturated, the addition of a ULMC method factor did not further improve the model fit (χ^2^/df = 0.83, CFI = 1.00, TLI = 1.00, SRMR = 0.017, RMSEA = 0.001 [0.001, 0.013]). These findings collectively indicate that the measurement model fits the data well and that no substantial common method bias exists in the present study.

**Table 3:** Fit indices for alternative measurement models.

Model	χ^2^/df	CFI	TLI	SRMR	RMSEA
One-factor	18.59	0.68	0.63	0.142	0.157 (0.150, 0.164)
Three-factor	0.82	1.00	1.00	0.017	0.001 (0.001, 0.012)
ULMC-factor	0.83	1.00	1.00	0.017	0.001 (0.001, 0.013)

One-factor model = all items loading on a single general factor; Three-factor model = each variable loading on its theoretical factor; ULMC-factor model = theoretical three factors plus an additional method factor.

### 4.4 Correlation analysis

The correlations among the key study variables are presented in Table 4. Green exercise was significantly and negatively correlated with both entrance anxiety (*r* = −0.31, *p* < 0.001) and suicidal ideation (*r* = −0.43, *p* < 0.001). Entrance anxiety was positively correlated with suicidal ideation (*r* = 0.43, *p* < 0.001). The values on the diagonal represent the square roots of the average variance extracted (AVE), all of which are greater than the inter-construct correlations, indicating satisfactory discriminant validity among the constructs.

**Table 4:** Correlations and discriminant validity among key variables.

Variable	Green exercise	Entrance anxiety	Suicidal ideation
Green exercise	0.73		
Entrance anxiety	−0.31^***^	0.73	
Suicidal ideation	−0.43^***^	0.43^***^	0.74

^***^p < 0.001. Values on the diagonal represent the square roots of the average variance extracted (AVE).

### 4.5 Multiple regression analysis

A hierarchical multiple regression analysis was conducted to examine the predictors of suicidal ideation. Model 1 included only the control variables (gender, family economic status, place of origin, and only-child status). Model 2 added the independent variable, green exercise, and Model 3 further incorporated the mediator, freshman anxiety. The standardized coefficients, standard errors, and model-fit indices are summarized in Table 5.

**Table 5:** Multiple regression analysis of predictors of suicidal ideation.

Variables	Model 1 (Controls only)		Model 2 (+Green exercise)		Model 3 (+Freshman anxiety)	
	β	SE	β	SE	β	SE
Control variables						
Gender	0.12^**^	0.68	0.09^*^	0.62	0.16^***^	0.59
Family economic status	−0.15^**^	0.50	−0.09^*^	0.46	−0.04	0.43
Place of origin	−0.11^*^	0.61	−0.06	0.56	−0.01	0.52
Only-child	0.01	0.68	0.01	0.62	0.01	0.57
Independent variable						
Green exercise			−0.42^***^	0.08	−0.31^***^	0.08
Mediator						
Freshman anxiety					0.36^***^	0.06
Model summary						
*R* ^2^	0.03		0.20		0.31	
Δ*R*^2^	–		0.17^***^		0.11^***^	
*F*	4.90^***^		35.18^***^		52.05^***^	

Standardized coefficients (β) are reported. ^*^p < 0.05, ^**^p < 0.01, ^***^p < 0.001.

In Model 2, the inclusion of green exercise significantly improved the explanatory power of the model (Δ*R*^2^ = 0.17, *p* < 0.001). Green exercise had a significant negative effect on suicidal ideation (β = −0.42, *p* < 0.001). When freshman anxiety was added in Model 3, it showed a significant positive effect on suicidal ideation (β = 0.36, *p* < 0.001), while the absolute value of the regression coefficient for green exercise decreased to −0.31 (*p* < 0.001). The total explained variance of suicidal ideation reached 31%.

### 4.6 Structural equation modeling analysis

To further verify the relationships among green exercise, entrance anxiety, and suicidal ideation, a structural equation model (SEM) was constructed. The model tested the direct and indirect effects of green exercise on suicidal ideation through entrance anxiety. The model demonstrated an excellent fit to the data: χ^2^/df = 0.82, CFI = 1.00, TLI = 1.00, SRMR = 0.017, and RMSEA = 0.001 (90% CI [0.001, 0.012]). All fit indices met the conventional criteria (χ^2^/df < 3, CFI and TLI > 0.90, SRMR < 0.08, RMSEA < 0.05), indicating that the hypothesized model adequately represented the observed data.

**Figure 2:**
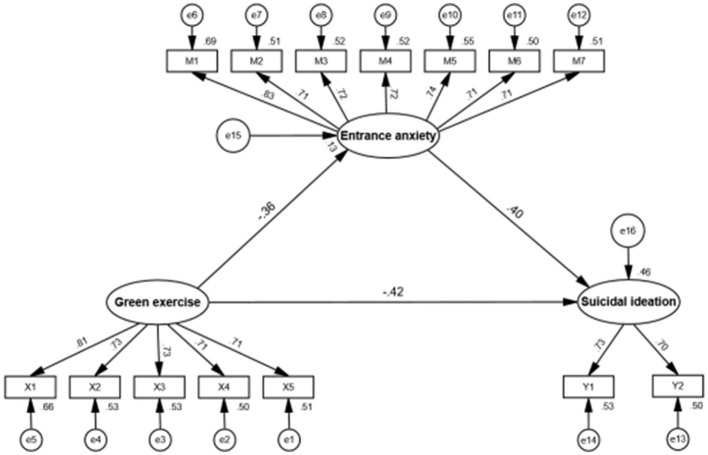
Standardized path coefficients of the structural model.

As shown in Figure 2, the standardized path coefficients revealed that green exercise had a significant negative effect on entrance anxiety (β = −0.36, *p* < 0.001) and suicidal ideation (β = −0.42, *p* < 0.001), thereby supporting H1 and H2. Entrance anxiety had a significant positive effect on suicidal ideation (β = 0.40, *p* < 0.001). These results suggest that entrance anxiety may serve as a mediating variable linking green exercise to suicidal ideation.

### 4.7 Mediation effect test

To further examine whether entrance anxiety mediated the relationship between green exercise and suicidal ideation, a bootstrapping mediation analysis was conducted using 5,000 resamples. Table 6 summarizes the total, direct, and indirect effects in the multiple mediator model.

**Table 6:** Total, direct and indirect effects in the multiple mediator model.

Path	β	Boot SE	*p*	Boot LLCI	Boot ULCI	Ratio (%)
Direct effect						
Green exercise → Suicidal ideation	−0.42	0.04	<0.001	−0.51	−0.34	73.68
Indirect effects						
Green exercise → Entrance anxiety → Suicidal ideation	−0.15	0.02	<0.001	−0.19	−0.10	26.32
**Total effect**	−0.57	0.04	<0.001	−0.64	−0.49	100

Boot LLCI, the lower bound of the 95% confidence interval. Boot ULCI, the upper limit of the 95% confidence interval (Percentile Bootstrap Method with Bias Correction). The Bootstrap sample size is set at 5,000.

The total effect of green exercise on suicidal ideation was significant (β = −0.57, *p* < 0.001, 95% CI [−0.64, −0.49]). After including entrance anxiety as a mediator, the direct effect of green exercise on suicidal ideation remained significant (β = −0.42, *p* < 0.001, 95% CI [−0.51, −0.34]), accounting for 73.68% of the total effect. The indirect effect of green exercise on suicidal ideation through entrance anxiety was also significant (β = −0.15, *p* < 0.001, 95% CI [−0.19, −0.10]), accounting for 26.32% of the total effect. The confidence intervals for both effects did not include zero, indicating a significant partial mediation effect of entrance anxiety between green exercise and suicidal ideation, thus supporting H3.

## 5 Discussion

### 5.1 Overview of the findings

This study examined the mechanism through which green exercise influences suicidal ideation among college freshmen and tested the mediating role of entrance anxiety. Based on stress–coping theory and perspectives consistent with Attention Restoration Theory (ART), the findings supported the proposed conceptual model.

Specifically, participation in green exercise was significantly associated with lower levels of entrance anxiety and suicidal ideation, indicating that physical activity in natural environments is related to better psychological adjustment during the university transition period. Students who engaged in green exercise more frequently reported fewer anxiety-related symptoms and a lower tendency toward suicidal thoughts, which is consistent with prior research emphasizing the stress-buffering and restorative functions of natural settings (Liu et al., 2025; Jimenez et al., 2021).

In addition, entrance anxiety showed a significant mediating effect between green exercise and suicidal ideation. This pattern suggests that anxiety reduction is a plausible psychological pathway linking green exercise to suicidal ideation. Overall, the evidence supports the study hypotheses: (H1) green exercise is negatively associated with suicidal ideation; (H2) green exercise is negatively associated with entrance anxiety; and (H3) entrance anxiety mediates the relationship between green exercise and suicidal ideation. In sum, green exercise was found to be associated with suicidal ideation both directly and indirectly through alleviating entrance anxiety, providing an empirically supported framework for understanding how nature-based activity may promote freshmen's mental health.

### 5.2 The relationship between green exercise and suicidal ideation

The results showed a significant negative association between green exercise and suicidal ideation, supporting H1 and further adding empirical evidence that nature-based physical activity is related to a lower risk of suicide-related thoughts. This suggests that green exercise may function as a protective behavioral correlate during the early stage of university entry.

From the perspective of the stress-buffering model, positive coping behaviors and environmental resources can reduce the negative psychological impact of stressors (Bekiros et al., 2022). Green exercise integrates physical activity with nature exposure, which may jointly facilitate stress management and emotion regulation. For freshmen facing academic uncertainty and social adaptation pressures, regular participation in green exercise may help reduce rumination, attenuate stress-related physiological arousal, and promote more adaptive coping responses, thereby decreasing negative affect and suicidal ideation.

This association is also broadly consistent with the perspectives of Attention Restoration Theory (ART) and the biophilia hypothesis, which propose that exposure to natural environments can restore depleted attentional resources and elicit more positive affective experiences (Jimenez et al., 2021; Gaekwad et al., 2022). Natural elements (e.g., greenery, sunlight, and open spaces) may facilitate relaxation and psychological recovery, thereby counteracting fatigue and psychological distress that may contribute to suicidal thinking. Overall, the present study suggests that green exercise is not only a leisure activity but may also represent an accessible and less stigmatizing behavioral approach associated with reduced suicidal ideation among freshmen, providing empirical support for incorporating ecological and behavioral factors into mental health promotion in higher education.

### 5.3 The mediating role of entrance anxiety

This study further found that entrance anxiety partially mediated the relationship between green exercise and suicidal ideation, supporting H3. This indicates that the association between green exercise and suicidal ideation is not only direct but is also partly achieved through an indirect pathway of anxiety reduction.

According to stress–coping theory, individuals are more likely to experience anxiety and other stress responses when they perceive environmental demands to exceed their coping resources (Lazarus and Folkman, 1984; Folkman, 2013). During the transition into university, freshmen may face academic pressure, social adaptation demands, and separation from familiar environments, thereby increasing entrance anxiety. Elevated anxiety may further intensify self-denial and rigid negative thinking patterns, which can increase the risk of suicidal ideation. Our findings provide empirical support for this pathway, suggesting that entrance anxiety may serve as a proximal psychological mechanism linking green exercise to suicidal ideation.

Green exercise may reduce entrance anxiety through the combined effects of “exercise-related benefits + nature exposure.” On the one hand, based on ART-related perspectives, natural environments may help individuals recover from attentional fatigue and reduce emotional tension (Jimenez et al., 2021; Zhang et al., 2024). On the other hand, physical activity stimulates endorphin release and enhances self-efficacy, thereby strengthening individuals' perceived capacity to manage stress (Hossain et al., 2024). Together, these processes may help students appraise transition-related stressors as more controllable and manageable, thus reducing entrance anxiety.

### 5.4 Theoretical implications

By focusing on college freshmen as a high-stress transitional population, this study connects green exercise to suicidal ideation through a parsimonious mediation model and highlights entrance anxiety as a key mechanism, thereby contributing to the relevant literature. The findings complement stress–vulnerability perspectives by suggesting that green exercise, as a behavioral–environmental factor, is associated with lower suicidal ideation and that this association is partly explained by reduced anxiety during the transition period. In addition to work emphasizing proximal cognitive and emotional vulnerabilities, the present results point to potentially meaningful distal protective correlates in the campus-life context (Neacsiu et al., 2018).

The findings are also compatible, to some extent, with ART in that contact with nature may facilitate psychological recovery and reduce distress. Importantly, because this study did not assess ART's four characteristic components (Being away, Extent, Fascination, and Compatibility) using an ART scale, the results should be interpreted as providing support for ART from a particular perspective rather than extending ART or positioning it as a foundational explanatory model. Future studies could directly measure ART dimensions to test ART mechanisms more rigorously, especially in relation to anxiety reduction and suicide-related cognition.

More broadly, identifying entrance anxiety as a mediator integrates environmental psychology with stress–coping theory and underscores the role of emotion regulation processes in linking nature-based behavior to suicidal ideation. Overall, the results support a focused, mechanism-oriented interpretation: green exercise is associated with suicidal ideation partly through alleviating entrance anxiety, which may be particularly relevant during the university transition period.

### 5.5 Practical implications

The findings have practical implications for mental health promotion in higher education. Green exercise was associated with lower suicidal ideation, and this association operated both directly and indirectly through reduced entrance anxiety. These results suggest that nature-based physical activity may be a feasible supplement to freshman-oriented mental health support.

Universities may consider increasing opportunities for green exercise during the first semester, when entrance anxiety is often more pronounced. Examples include organized outdoor walking/jogging activities, group sports in campus green spaces, and nature-based physical education modules. Such options are typically low-cost and accessible, and they may also complement traditional psychological services in a relatively non-stigmatizing manner.

Because green exercise depends on the availability of natural spaces, campus administrators can further support routine outdoor activity by improving the accessibility and usability of green areas (e.g., safe walking routes, open lawns, and small gardens). Emphasizing convenience and safety may help translate students' intentions into stable participation patterns.

Given the mediating role of entrance anxiety, programs that combine transition support with outdoor activity may be especially relevant for freshmen. Orientation programs or freshman seminars, for instance, could integrate brief psychoeducation on stress management with guided outdoor activities, encouraging students to adopt green exercise as a coping strategy during the adjustment period.

Overall, the practical value of this study lies in showing that green exercise is an accessible and ecologically grounded approach that may help reduce entrance anxiety and suicidal ideation. Embedding nature-based exercise within university culture may support psychological resilience and emotional balance among college students.

### 5.6 Limitations and future directions

Despite its theoretical and practical significance, several considerations should be noted, and these also point to directions for future research.

The cross-sectional design limits conclusions about temporal ordering and causality among green exercise, entrance anxiety, and suicidal ideation. Although SEM and bootstrapped mediation analyses provided statistical support for the proposed relationships, longitudinal or intervention studies are needed to test whether changes in green exercise precede changes in anxiety and suicidal ideation over time.

This research was conducted from a subjective evaluation perspective, using self-report questionnaires to capture perceived green exercise participation, entrance anxiety, and suicidal ideation. Self-report approaches are appropriate for internal psychological experiences; however, responses may be influenced by common method variance, social desirability, and individual interpretation. Future work could strengthen robustness through multi-method and multi-source assessments, such as combining self-reports with behavioral indicators (e.g., objectively recorded activity levels), physiological measures (e.g., cortisol, heart rate variability), or digital activity tracking, thereby providing convergent evidence across multiple dimensions.

The study intentionally focused on first-year university students, given that entrance anxiety is most salient during the initial transition into university and central to the proposed mechanism. Accordingly, the current conclusions are most applicable to the freshman transition context. Future studies may extend the model to other student groups (e.g., upper-year students) and broader populations (e.g., adolescents, working adults, or clinical samples) to examine whether the observed associations and mechanisms are stable across contexts or vary by developmental stage and life setting.

Demographic characteristics were reported descriptively, but variables such as gender, personality traits, exercise frequency, and the quality of natural environments were not modeled as moderators. These factors may influence the magnitude or direction of the observed associations. Future research could use moderation or moderated mediation approaches (e.g., multi-group SEM or interaction-based models) to test heterogeneity and clarify for whom and under what conditions green exercise may be most protective.

Finally, although this study tested entrance anxiety as a single mediator, suicidal ideation is multifaceted and may also be influenced by other processes (e.g., loneliness, meaning in life, or perceived social support). Future research could extend the current model by examining additional mediators or multi-pathway frameworks to provide a more comprehensive account of how green exercise relates to suicidal cognition.

## 6 Conclusion

In conclusion, this study provides empirical evidence that green exercise functions as a protective factor against suicidal ideation among college freshmen, both directly and indirectly through the reduction of entrance anxiety. By integrating stress-coping theory, attention restoration theory, and the environmental psychology perspective, the study establishes a comprehensive framework linking behavioral engagement in natural environments with psychological resilience. The findings highlight that contact with nature through physical activity not only enhances emotional regulation but also alleviates transitional anxiety, thereby reducing the cognitive vulnerability that underlies suicidal thinking. These insights advance theoretical understanding of the interplay between environment, behavior, and mental health, while offering practical guidance for universities to promote nature-based exercise as a sustainable strategy for preventing psychological distress and fostering wellbeing among students.

## Data Availability

The original contributions presented in the study are included in the article/Supplementary material, further inquiries can be directed to the corresponding author.

## References

[B1] Ahindru A. Patil P. Agrawal V. (2023). Role of physical activity on mental health and well-being: a review. Cureus 15:e33475. doi: 10.7759/cureus.33475 36756008 PMC9902068

[B2] Ahmad N. Alias F. A. Hamat M. Mohamed S. A. (2024). “Reliability analysis: Application of Cronbach's alpha in research instruments,” in Pioneering the Future: Delving into e-Learning's Landscape, eds. Kadar R. Wan Mohammad W. A. and Mydin A. M. (Permatang Pauh: Unit Penerbitan Jabatan Sains Komputer & Matematik (JSKM), Bahagian Hal Ehwal Akademik (BHEA), Universiti Teknologi MARA Cawangan Pulau Pinang), 114.

[B3] Anderson E. Shivakumar G. (2013). Effects of exercise and physical activity on anxiety. Front. Psychiatry 4:27. doi: 10.3389/fpsyt.2013.00027 23630504 PMC3632802

[B4] Aras S. G. Runyon J. R. Kazman J. B. Thayer J. F. Sternberg E. M. Deuster P. A. (2024). Is greener better? Quantifying the impact of a nature walk on stress reduction using HRV and saliva cortisol biomarkers. Int. J. Environ. Res. Public Health 21:1491. doi: 10.3390/ijerph21111491 39595758 PMC11594215

[B5] Asfaw H. Yigzaw N. Yohannis Z. Fekadu G. Alemayehu Y. (2020). Prevalence and associated factors of suicidal ideation and attempt among undergraduate medical students of Haramaya University, Ethiopia: a cross-sectional study. PLoS ONE 15:e0236398. doi: 10.1371/journal.pone.0236398 32785295 PMC7423400

[B6] Barton J. Pretty J. (2010). What is the best dose of nature and green exercise for improving mental health? A multi-study analysis. Environ. Sci. Technol. 44, 3947–3955. doi: 10.1021/es903183r 20337470

[B7] Beck A. T. Steer R. A. Beck J. S. Newman C. F. (1993). Hopelessness, depression, suicidal ideation, and clinical diagnosis of depression. Suicide Life Threatening Behav. 23, 139–145. doi: 10.1111/j.1943-278X.1993.tb00378.x 8342213

[B8] Bekiros S. Jahanshahi H. Munoz-Pacheco J. M. (2022). A new buffering theory of social support and psychological stress. PLoS ONE 17:e0275364. doi: 10.1371/journal.pone.0275364 36223401 PMC9555651

[B9] Bramwell R. C. Streetman A. E. Besenyi G. M. (2023). The effect of outdoor and indoor group exercise classes on psychological stress in college students: a pilot study with randomization. Int. J. Exerc. Sci. 16, 1012–1024. doi: 10.70252/EERP4920 37650002 PMC10464750

[B10] Bratman G. N. Anderson C. B. Berman M. G. Cochran B. de Vries S. Flanders J. . (2019). Nature and mental health: an ecosystem service perspective. Sci. Adv. 5:eaax0903. doi: 10.1126/sciadv.aax0903 31355340 PMC6656547

[B11] Bratman G. N. Hamilton J. P. Hahn K. S. Daily G. C. Gross J. J. (2015). Nature experience reduces rumination and subgenual prefrontal cortex activation. Proc. Natl. Acad. Sci. U.S.A. 112, 8567–8572. doi: 10.1073/pnas.1510459112 26124129 PMC4507237

[B12] Bratman G. N. Young G. Mehta A. Lee Babineaux I. Daily G. C. Gross J. J. (2021). Affective benefits of nature contact: the role of rumination. Front. Psychol. 12:643866. doi: 10.3389/fpsyg.2021.643866 33776870 PMC7988226

[B13] Cecchin H. F. G. da Costa H. E. R. Pacheco G. R. de Valencia G. B. Murta S. G. (2024). Risk factors for suicidal ideation in Brazilian university students: a mixed methods study. Trends Psychol. 32. doi: 10.1007/s43076-024-00402-2

[B14] Cheung G. W. Cooper-Thomas H. D. Lau R. S. Wang L. (2024). Reporting reliability, convergent and discriminant validity with structural equation modeling: a review and best-practice recommendations. Asia Pac. J. Manage. 41, 745–783. doi: 10.1007/s10490-023-09871-y

[B15] Coventry P. A. Brown J. E. Pervin J. Brabyn S. Pateman R. Breedvelt J. . (2021). Nature-based outdoor activities for mental and physical health: systematic review and meta-analysis. SSM Popul. Health 16:100934. doi: 10.1016/j.ssmph.2021.100934 34646931 PMC8498096

[B16] Craft L. L. Perna F. M. (2004). The benefits of exercise for the clinically depressed. Prim. Care Companion J. Clin. Psychiatry 6, 104–111. doi: 10.4088/PCC.v06n0301 15361924 PMC474733

[B17] De Brito J. N. Pope Z. C. Mitchell N. R. Schneider I. E. Larson J. M. Horton T. H. . (2020). The effect of green walking on heart rate variability: a pilot crossover study. Environ. Res. 185:109408. doi: 10.1016/j.envres.2020.109408 32220745 PMC7877549

[B18] Folkman S. (2013). “Stress: appraisal and coping,” in Encyclopedia of Behavioral Medicine, eds. Gellman M. D. and Turner J. R. (New York, NY: Springer), 1913–1915. doi: 10.1007/978-1-4419-1005-9_215

[B19] Gaekwad J. S. Sal Moslehian A. Roös P. B. Walker A. (2022). A meta-analysis of emotional evidence for the biophilia hypothesis and implications for biophilic design. Front. Psychol. 13:750245. doi: 10.3389/fpsyg.2022.750245 35693493 PMC9186521

[B20] Gladwell V. F. Brown D. K. Wood C. Sandercock G. R. H. Barton J. L. (2013). The great outdoors: how a green exercise environment can benefit all. Extreme Physiol. Med. 2:3. doi: 10.1186/2046-7648-2-3 23849478 PMC3710158

[B21] Hager N. M. Judah M. R. Milam A. L. (2022). Loneliness and depression in college students during the COVID-19 pandemic: the role of boredom and repetitive negative thinking. Int. J. Cogn. Ther. 15, 134–152. doi: 10.1007/s41811-022-00135-z 35432692 PMC8990489

[B22] Hair J. F. (2011). “Multivariate data analysis: an overview,” in International Encyclopedia of Statistical Science, ed. M. Lovric (Berlin, Heidelberg: Springer), 904–907. doi: 10.1007/978-3-642-04898-2_395

[B23] Han X. Li H. Xiao C. (2025). Physical activity enhances college students' mental health through social adaptability and exercise behavior chain mediation. Sci. Rep. 15:21127. doi: 10.1038/s41598-025-07791-z 40594661 PMC12214962

[B24] He Y. Yang T. He C. (2025). The relationship between college students' suicidal ideation and rejection sensitivity: a network analysis. BMC Public Health 25:343. doi: 10.1186/s12889-025-23877-9 39871231 PMC11773858

[B25] Henriksson M. Wall A. Nyberg J. Adiels M. Lundin K. Bergh Y. . (2022). Effects of exercise on symptoms of anxiety in primary care patients: a randomized controlled trial. J. Affect. Disord. 297, 26–34. doi: 10.1016/j.jad.2021.10.006 34644619

[B26] Hossain M. N. Lee J. Choi H. Kwak Y. S. Kim J. (2024). The impact of exercise on depression: how moving makes your brain and body feel better. Phys. Activity Nutr. 28, 43–51. doi: 10.20463/pan.2024.0015 39097997 PMC11298280

[B27] Howlader S. Abedin S. Rahman M. M. (2024). Social support, distress, stress, anxiety, and depression as predictors of suicidal thoughts among selected university students in Bangladesh. PLoS Global Public Health 4:e0002924. doi: 10.1371/journal.pgph.0002924 38626087 PMC11020522

[B28] Hu F. H. Zhao D. Y. Fu X. L. Zhang W. Q. Tang W. Hu S. Q. . (2023). Effects of social support on suicide-related behaviors in patients with severe mental illness: a systematic review and meta-analysis. J. Affect. Disord. 328, 324–333. doi: 10.1016/j.jad.2023.02.070 36813042

[B29] Huang F. Lu W. Zhao X. Li N. Zhao T. Guo S. . (2024). Suicidal ideation in medical students of Hebei province: prevalence and associated factors. Front. Psychiatry 15:1398668. doi: 10.3389/fpsyt.2024.1398668 39140111 PMC11319246

[B30] Huang Y. Zhang L. Li N. Chen Y. Wang X. (2024). The relationship between social media addiction and anxiety among college students: the mediating role of sleep procrastination and the moderating role of perceived stress. Modern Prev. Med. 51, 3756–3761. doi: 10.20043/j.cnki.MPM.202407048

[B31] Hunt J. Eisenberg D. (2010). Mental health problems and help-seeking behavior among college students. J. Adolescent Health 46, 3–10. doi: 10.1016/j.jadohealth.2009.08.008 20123251

[B32] Jeffrey J. Hoogland J. (1998). Robustness studies in covariance structure modeling: an overview and a meta-analysis. Sociol. Methods Res. 26, 329–367. doi: 10.1177/0049124198026003003

[B33] Jimenez M. P. DeVille N. V. Elliott E. G. Schiff J. E. Wilt G. E. Hart J. E. . (2021). Associations between nature exposure and health: a review of the evidence. Int. J. Environ. Res. Public Health 18:4790. doi: 10.3390/ijerph18094790 33946197 PMC8125471

[B34] Jones T. (2024). Adjustment Issues in First-Year College Students (Honors research project No. 1827). Williams Honors College. Availble online at: https://ideaexchange.uakron.edu/honors_research_projects/1827 (Accessed October 30, 2025).

[B35] Kaplan S. (1995). The restorative benefits of nature: toward an integrative framework. J. Environ. Psychol. 15, 169–182. doi: 10.1016/0272-4944(95)90001-2

[B36] Klonoff-Cohen H. S. Cohen A. Gobin R. L. Polavarapu M. Allen R. Reddy S. . (2024). Suicide ideation and self-harm behaviors in first-year dormitory students at a public Midwestern university: a pilot study. Chronic Stress 8:24705470241259939. doi: 10.1177/24705470241259939 38846597 PMC11155327

[B37] Klonsky E. D. Pachkowski M. C. Shahnaz A. May A. M. (2021). The three-step theory of suicide: description, evidence, and some useful points of clarification. Prev. Med. 152(Pt 1):106549. doi: 10.1016/j.ypmed.2021.106549 34538372

[B38] Kock F. Berbekova A. Assaf A. G. (2021). Understanding and managing the threat of common method bias: detection, prevention, and control. Tour. Manage. 86:104330. doi: 10.1016/j.tourman.2021.104330

[B39] Lahart I. Darcy P. Gidlow C. Calogiuri G. (2019). The effects of green exercise on physical and mental wellbeing: a systematic review. Int. J. Environ. Res. Public Health 16:1352. doi: 10.3390/ijerph16081352 30991724 PMC6518264

[B40] Lazarus R. S. Folkman S. (1984). Stress, Appraisal, and Coping. New York, NY: Springer Publishing Company.

[B41] Li W. Huo S. Yin F. (2024). The differences in symptom networks of depression, anxiety, and sleep in college students with different stress levels. BMC Public Health 24:3609. doi: 10.1186/s12889-024-21161-w 39736526 PMC11684240

[B42] Liu H. Wang Y. He Q. Li Y. Zhang J. Cahen Y. . (2025). Assessing the restorative effects of campus greenness on student depression: a comparative study across three distinct university campus types in Macau. BMC Public Health 25:907. doi: 10.1186/s12889-025-21356-9 40050861 PMC11887352

[B43] Loureiro A. Veloso S. (2017). “Green exercise, health and well-being,” in Handbook of Environmental Psychology and Quality of Life Research, eds. Fleury-Bahi G. Pol E. and Navarro O. (Cham: Springer), 155–172. doi: 10.1007/978-3-319-31416-7_8

[B44] Mackay G. J. Neill J. T. (2010). The effect of “green exercise” on state anxiety and the role of exercise duration, intensity, and greenness: a quasi-experimental study. Psychol. Sport Exerc. 11, 238–245. doi: 10.1016/j.psychsport.2010.01.002

[B45] Neacsiu A. D. Fang C. M. Rodriguez M. Rosenthal M. Z. (2018). Suicidal behavior and problems with emotion regulation. Suicide Life Threatening Behav. 48, 52–74. doi: 10.1111/sltb.12335 28261853

[B46] Okechukwu F. O. Ogba K. T. Nwufo J. I. Nwoke I. A. Nwankwo A. E. (2022). Academic stress and suicidal ideation: moderating roles of coping style and resilience. BMC Psychiatry 22:546. doi: 10.1186/s12888-022-04063-2 35962365 PMC9373522

[B47] Osman A. Gutierrez P. M. Kopper B. A. Barrios F. X. Chiros C. E. (1998). The positive and negative suicide ideation inventory: development and validation. Psychol. Rep. 82(3 Pt 1), 783–793. doi: 10.2466/pr0.1998.82.3.783 9676490

[B48] Park C. McClure Fuller M. Echevarria T. M. Nguyen K. Perez D. Masood H. . (2023). A participatory study of college students' mental health during the first year of the COVID-19 pandemic. Front. Public Health 11:1116865. doi: 10.3389/fpubh.2023.1116865 37026129 PMC10070728

[B49] Preacher K. J. Hayes A. F. (2008). Asymptotic and resampling strategies for assessing and comparing indirect effects in multiple mediator models. Behav. Res. Methods 40, 879–891. doi: 10.3758/BRM.40.3.879 18697684

[B50] Pretty J. Griffin M. Sellens M. H. Pretty C. G. (2003). Green Exercise: Complementary Roles of Nature, Exercise and Diet in Physical and Emotional Well-being and Implications for Public Health Policy. Colchester: University of Essex, Centre for Environment and Society.

[B51] Pretty J. Peacock J. Sellens M. Griffin M. (2005). The mental and physical health outcomes of green exercise. Int. J. Environ. Health Res. 15, 319–337. doi: 10.1080/09603120500155963 16416750

[B52] Saricali M. Satici S. A. Satici B. Gocet-Tekin A. Griffiths A. (2022). Fear of COVID-19, mindfulness, humor, and hopelessness: a multiple mediation analysis. Int. J. Ment. Health Addict. 20, 2151–2164. doi: 10.1007/s11469-020-00419-5 33230394 PMC7676415

[B53] Satyander and Kumar S. (2024). Suicidal ideation and cognitive distortions among undergraduate students. Educ. Admin. Theory Prac. 30, 11239–11245. doi: 10.53555/kuey.v30i5.4922

[B54] Steinberg L. J. Mann J. J. (2020). Abnormal stress responsiveness and suicidal behavior: a risk phenotype. Biomark. Neuropsychiatry 2:100011. doi: 10.1016/j.bionps.2020.100011

[B55] Tang D. D. Wen Z. L. (2020). Common method bias testing: Problems and recommendations. Psychol. Sci. 43, 215–223. doi: 10.16719/j.cnki.1671-6981.20200130

[B56] Thompson Coon J. Boddy K. Stein K. Whear R. Barton J. Depledge M. H. (2011). Does participating in physical activity in outdoor natural environments have a greater effect on physical and mental wellbeing than physical activity indoors? A systematic review. Environ. Sci. Technol. 45, 1761–1772. doi: 10.1021/es102947t 21291246

[B57] Twohig-Bennett C. Jones A. (2018). The health benefits of the great outdoors: a systematic review and meta-analysis of greenspace exposure and health outcomes. Environ. Res. 166, 628–637. doi: 10.1016/j.envres.2018.06.030 29982151 PMC6562165

[B58] Van Heeringen K. (2012). “Stress–diathesis model of suicidal behavior,” in The Neurobiological Basis of Suicide, ed. Y. Dwivedi (Boca Raton, FL: CRC Press/Taylor and Francis), Chap. 6. 23035289

[B59] Wen L. Zhang L. Zhu L. (2024). Depression and suicidal ideation among Chinese college students during the COVID-19 pandemic: the mediating roles of chronotype and sleep quality. BMC Psychiatry 24:583. doi: 10.1186/s12888-024-06027-0 39192231 PMC11348737

[B60] Wicks C. Barton J. Orbell S. Andrews L. (2022). Psychological benefits of outdoor physical activity in natural versus urban environments: a systematic review and meta-analysis of experimental studies. Appl. Psychol. Health Well-Being 14, 1037–1061. doi: 10.1111/aphw.12353 35259287 PMC9544808

[B61] Wood C. Barron D. Smyth N. (2019). The current and retrospective intentional nature exposure scales: development and factorial validity. Int. J. Environ. Res. Public Health 16:4443. doi: 10.3390/ijerph16224443 31726784 PMC6888483

[B62] Yang T. He Y. Wu L. Ren L. Lin J. Wang C. . (2023). The relationships between anxiety and suicidal ideation and between depression and suicidal ideation among Chinese college students: a network analysis. Heliyon 9:e20938. doi: 10.1016/j.heliyon.2023.e20938 37876446 PMC10590950

[B63] Zhang J. Jin J. Liang Y. (2024). The impact of green space on university students' mental health: the mediating roles of solitude competence and perceptual restoration. Sustainability 16:707. doi: 10.3390/su16020707

[B64] Zhang T. Kong J. (2025). How does exercise regulate the physiological responses of post-traumatic stress disorder? The crosstalk between oxidative stress and the hypothalamic–pituitary–adrenal axis. Front. Physiol. 16:1567603. doi: 10.3389/fphys.2025.1567603 41018015 PMC12466154

